# Hypertension in Children: Narrative Review of Epidemiology, Outcome and Target Organ Damage

**DOI:** 10.3390/children13060831

**Published:** 2026-06-18

**Authors:** Joseph Mahgerefteh

**Affiliations:** Division of Pediatric Cardiology, Department of Pediatrics, Kravis Children’s Hospital, Icahn School of Medicine at Mount Sinai, New York, NY 10029, USA; joseph.mahgerefteh@mssm.edu

**Keywords:** hypertension, pediatric, target organ damage, epidemiology, cardiovascular

## Abstract

**Highlights:**

**What are the main findings?**

- Hypertension is prevalent in children and, if left untreated, may contribute to premature atherosclerosis.
- Treatment of hypertension in children is associated with improved intermediate outcomes.

**What are the implications of the main findings?**

- Optimized screening and diagnostic algorithms for pediatric hypertension identify children at higher risk of premature heart disease.
- Optimized pediatric hypertension treatment protocols may improve long term cardiovascular outcomes.

**Abstract:**

Hypertension is the leading risk factor for cardiovascular disease. Epidemiologic studies have demonstrated that pediatric hypertension may increase the risk of premature heart disease. Pediatric hypertension affects about 4% of children and clusters with other risk factors and social disparities in health. In addition to observed target organ damage, there is evidence for tracking blood pressure from childhood to adulthood. Ambulatory blood pressure monitoring is the recommended method for diagnosis, and echocardiography is used to assess target organ damage. A diagnostic workup in children depends on the age at presentation, severity of hypertension, diurnal pattern, evidence of target organ damage, and response to treatment. Treatment follows a similar framework to adult hypertension and studies demonstrate improvement in intermediate outcomes with treatment. However, further studies are needed to establish benefit in hard outcomes. This review focuses on studies evaluating the epidemiology of pediatric hypertension and its association with cardiovascular outcomes. Relevant domains included prevalence, blood pressure tracking, and cardiovascular sequelae. To compile data for this narrative review, a PubMed/MEDLINE database search was performed for studies published between 1997 and April 2026.

## 1. Introduction

Hypertension (HTN) is an important risk factor for cardiovascular disease (CVD) and premature mortality worldwide [1]. Since the publication of the first report of the Task Force on Blood Pressure (BP) in Children in 1977 [2], a substantial body of research and numerous publications have identified pediatric HTN as a risk factor for premature cardiovascular disease [3]. Epidemiologic studies suggest that the global prevalence of pediatric HTN has increased over time [4]. This increasing prevalence, in addition to studies demonstrating that BP tracks from childhood to adulthood, highlights the importance of BP evaluation and management in children [5]. There is a significant focus on intermediate outcomes in pediatric HTN research. Target organ damage, as an intermediate outcome in pediatric HTN, has been the primary focus of short-term studies and includes structural and functional cardiovascular alterations [6,7,8,9]. The clustering of other risk factors such as obesity, dyslipidemia, and diabetes with pediatric HTN significantly increases cardiovascular risk beyond the additive effects of individual risk factors and warrants further focus on evaluation and treatment of HTN in children [10].

This review summarizes current evidence on pediatric HTN as a cardiovascular risk factor, highlighting epidemiological data and long-term cardiovascular outcomes. Additionally, we discuss target organ damage, diagnostic strategies and management approaches while highlighting future research directions. It also highlights the knowledge gaps regarding optimal diagnostic criteria and discusses the lack of evidence for improvement in hard outcomes with the treatment of HTN in children.

## 2. Epidemiology

Estimates regarding the global prevalence of pediatric HTN depend on the definitions used. The American Academy of Pediatrics (AAP) defines HTN based on the 95th percentile for BP according to age, height, and sex. However, the AAP emphasizes the importance of at least three different measurements for confirmation of diagnosis. Based on these criteria, two recent systematic reviews report a global prevalence of 3.89–4.28% in children [4,11]. Using ambulatory BP monitoring in addition to clinic measurements for diagnosis, sustained HTN affects 6.67% of children [4]. The severity of HTN is important because higher stages of HTN are associated with a greater risk of CVD. Stage 2 HTN is less common, and its estimated global prevalence is 0.67% [11]. Globally, between 2000 and 2020 the prevalence of pediatric HTN doubled but US-specific NHANES data suggest stabilization or decline in recent years (2015–2023) [12]. Geographic variation in prevalence is largely attributable to differences in obesity prevalence, dietary patterns, physical activity, and diagnostic criteria. The relationship between obesity and HTN prevalence is dose-dependent and specifically seen with central obesity. The prevalence of HTN in obese children is 16.35–18.77% versus 2.44–2.57% in children with normal BMI (8-fold increase). The interaction among obesity, HTN, and social determinants of health warrants special attention as clustering of these risk factors poses a significant risk. The All of Us study, which included 219,700 participants, showed that a one-standard-deviation increase in neighborhood median income was linked to a 12% reduction in the odds of undiagnosed HTN [13]. Multiple interconnected pathways are responsible for the relationship between social determinants of health and pediatric HTN. These include chronic stress, epigenetic reprogramming, nutrition, and environmental exposures and their effects on sympathetic and inflammatory pathways [14]. The impact of interventions targeting these pathways is beyond the scope of this review; however, several promising interventions have been reported.

Studies assessing the association between childhood BP and adult HTN have demonstrated moderate BP tracking over time. A recent analysis of the Young Finns study demonstrated that children with elevated blood pressure are at a 1.45-fold-increased risk of developing adult HTN [15]. This study demonstrated higher risk in children with more severe forms of BP abnormality. This, in addition to the higher risk of target organ damage and adverse cardiovascular outcomes in childhood and adulthood, highlights the importance of diagnosing HTN in children [3,5].

The most recent statement from the United States Preventive Services Task Force in 2020 stated that current evidence was insufficient to recommend routine screening for elevated BP in children [16]. One of the prominent issues raised is the limited accuracy of clinic BP measurement. Pediatric studies comparing triage BP measurements with standardized BP measurements obtained in pediatric HTN clinics have demonstrated poor agreement [17,18]. One study assessed a cohort of obese children and compared triage BP measurements with standardized BP measurements. This study demonstrated that there was a mean systolic BP difference of 8.7 mmHg (95% limits of agreement by Bland–Altman analysis: −16.66, +34.07 mmHg). Overall agreement in BP classification was 57.6% [17]. Studies in adults have evaluated the impact of arm position and other specific factors on BP measurement accuracy [19,20]. These studies highlight the importance of standardized BP measurement for accurate classification. Ambulatory blood pressure monitoring (ABPM) is the gold standard for measurement and diagnosis of HTN. When clinic BP measurements are combined with ABPM, four BP phenotypes can be identified. Identification of white-coat HTN (abnormal clinic BP with normal ABPM) and masked HTN (normal clinic BP with abnormal ABPM) is helpful in risk stratification and decisions to treat [21]. In a study assessing otherwise healthy adolescents with clinic BP between the 75th and 95th percentiles, the prevalence of masked HTN and white-coat HTN were 11% and 15%, respectively [22]. Although the prevalence of white-coat HTN among children referred for evaluation of elevated BP varies, one study assessing the cost-effectiveness of ABPM as the initial evaluation for children with clinic BP values in the stage 1 HTN range demonstrated significant cost savings [23]. On the other hand, ABPM in special high-risk groups with normal clinic BP demonstrated significantly improved detection of HTN in these populations. A systematic review demonstrated that the prevalence of masked HTN in children with a history of coarctation of the aorta, solid organ or stem cell transplant, chronic kidney disease and sickle cell disease is much higher than that of the general pediatric population [24].

## 3. Long-Term Cardiovascular Outcomes

While adult studies have demonstrated that HTN is one of the most preventable causes of death [1], studies directly assessing childhood BP and long-term cardiovascular outcomes remain limited (Table 1). The International Childhood Cardiovascular Cohort (i3C) Consortium published a study assessing the relationship between childhood risk factors including BP with long-term cardiovascular outcomes. The authors demonstrated that hazard ratios for fatal and non-fatal adult cardiovascular events among children with HTN were 2.04 and 2.31, respectively [25]. Another cohort study in Ontario, Canada, demonstrated that children with HTN who were followed for an average of 13.6 years exhibited a two-fold-higher risk of major adverse cardiovascular events compared to children with normal BP. This translates to one additional major adverse cardiovascular event for 417 child-years of follow-up [3]. This study did not demonstrate a difference in cardiovascular mortality. Another recent study evaluating the long-term impact of HTN and elevated BP diagnosed at 7 years of age demonstrated a hazard ratio for cardiovascular mortality of 1.40 and 1.48, respectively. This model was adjusted for childhood body mass index, maternal race, education, and marital status, and study site [26].

Adolescents with elevated blood pressure (BP) or HTN are at significantly increased risk of developing or maintaining HTN in adulthood. This association varies depending on age at initial evaluation, the severity of BP elevation, and the duration of follow-up. A meta-analysis of 11 studies demonstrated that children with elevated BP have approximately a two-fold-higher risk of developing HTN as adults [27]. Another study assessing tracking and transition probability concluded that adolescents with HTN have a 16–44% chance of reverting to normal BP in adulthood [15]. Another study assessing BP tracking over 38 years of follow-up demonstrated that being in the highest versus the lowest childhood systolic BP quartile is associated with a higher risk of adult HTN (female hazard ratio, 3.85; male, 2.66) [28].

Published studies have not specifically assessed the impact of childhood HTN on life expectancy. Adult studies report an estimated 5 years of reduced life expectancy related to HTN diagnosed at 50 years of age [29]. These data, in addition to previously discussed data about tracking, suggest that persistent HTN from childhood into adulthood may have a substantial adverse impact on life expectancy, although this has not been directly evaluated.

It is important to note that most studies evaluating the long-term cardiovascular outcomes of pediatric HTN are observational in nature. Consequently, the ability to determine the independent contribution of pediatric HTN to cardiovascular outcomes is limited by potential confounding factors such as obesity, obstructive sleep apnea, and genetic predisposition.

**Table 1:** Longitudinal studies of relationship between childhood BP and adult cardiovascular outcome.

Study	Design	Population/N	Follow-Up	Key Finding
i3C Consortium [25]	Prospective cohort consortium	~38,000 participants from 7 cohorts	Up to 35+ years	Combined childhood risk factor z-score associated with adult CV events; SBP z-score HR of 1.30 (95% CI 1.14–1.47) for fatal events
Ontario Cohort [3]	Population-based retrospective cohort	~25,000 children with HTN diagnosis	Mean 13.6 years	2-fold-higher risk of MACE; 1 additional MACE per 417 child-years; no difference in CV death
BP at age 7 study [26]	Prospective cohort	~37,000 children evaluated at age 7	Decades	HR for CV mortality: 1.40 (HTN), 1.48 (elevated BP); adjusted for BMI, maternal race, education
Young Finns/Bogalusa (tracking) [28,30]	Prospective cohort	~3000–4000 per cohort	25–38 years	Elevated childhood BP → 1.45-fold risk of adult HTN; highest- vs. lowest-SBP-quartile HR of 2.66–3.85
Meta-analysis [27]	Meta-analysis of 11 studies	Pooled pediatric cohorts	Variable	~2-fold-higher risk of adult HTN in children with elevated BP

## 4. Cardiac Target Organ Damage in Youth

A large number of studies have assessed cardiac target organ damage in childhood HTN. These studies demonstrated structural and functional cardiac alterations secondary to HTN. A meta-analysis of studies published between 1974 and 2021 found that children with ambulatory HTN have a 4.69-fold-higher risk of developing left ventricular hypertrophy (LVH) [31]. Another meta-analysis reported that the prevalence of LVH in children with untreated HTN may be as high as 30% [32]. Even though there is no direct association between childhood LVH and adult cardiovascular outcomes, as mentioned above several studies have demonstrated an association between pediatric HTN and adult CV outcomes. In addition, multiple longitudinal studies have demonstrated an association between pediatric HTN and adult LVH. The Bogalusa Heart Study demonstrated that adolescent HTN is associated with adult LVH and cardiac remodeling [33]. In adult studies, LVH and changes in left ventricular mass and geometry are associated with a higher risk of mortality. Importantly, regression of LVH with treatment is associated with improved cardiovascular outcomes [34]. Studies in children demonstrated that LVH regression can be achieved with appropriate pharmacologic treatment and lifestyle intervention [35,36]. Given the significant overlap between obesity and HTN, studies evaluating LV remodeling patterns have demonstrated a higher prevalence of eccentric hypertrophy versus concentric hypertrophy (19.1–24.4% versus 15.1–19.1%) [37,38]. The pathological interaction between obesity and HTN in addition to methodology of left ventricular mass normalization will affect these incidences. Further studies are needed to better differentiate between physiologic and pathological changes in growing children [39] but consistency with findings from adult studies suggests that there are pathologic changes that occur in pediatric HTN. Current pediatric HTN guidelines recommend obtaining an echocardiogram when initiating treatment for HTN. Repeat echocardiography is advised at 6–12-month intervals to assess for regression or progression of cardiac changes [40].

In addition to LVH, pediatric HTN has been associated with both diastolic dysfunction and subclinical systolic impairment. Meta-analyses comparing children with HTN to normotensive controls demonstrate significant alterations in diastolic parameters, including reduced E/A ratios and elevated E/e′ values, findings consistent with early pathologic myocardial changes. Furthermore, global longitudinal strain—a sensitive marker of subclinical systolic function— has been shown to be reduced in children with HTN [6,7]. It is important to recognize that pediatric HTN rarely causes overt systolic dysfunction. In a cohort of children with HTN-related cardiac dysfunction who achieved BP control, the majority (81%) recovered. This recovery was most frequent in infants (92%) [41]. This study demonstrates that hypertensive cardiomyopathy in childhood is often reversible when afterload is corrected. However, the impact of HTN on systolic function varies significantly with age. In infants, the myocardial response to elevated afterload is typically more pronounced but also more reversible, often resolving with effective BP control or correction of the underlying cause. Hypothetically, this difference may be related to developmental differences in myocardial structure and metabolism, including higher water content, fewer mitochondria, and reduced energy reserves in infants. In contrast, adolescents tend to exhibit subtler systolic impairment, which may not fully normalize even after afterload reduction. This may be related to progressive myocardial fibrosis and scarring. The detection of these abnormalities allows for risk stratification and targeted intervention before irreversible damage occurs.

## 5. Early Vascular and Microvascular Changes and Markers

Studies have demonstrated that childhood HTN is associated with increased carotid intima–media thickness, elevated pulse wave velocity, endothelial dysfunction, and abnormalities of the retinal microvasculature. A meta-analysis of studies including children with HTN diagnosed by ambulatory blood pressure monitoring demonstrated a mean increase in carotid intima–media thickness of 0.04 mm and pulse wave velocity of 0.39 m/s between the hypertensive and normotensive groups [31]. These associations were found to be relatively weak in this meta-analysis. However, studies have shown that changes in carotid intima–media thickness may be reversible with treatment [42]. Endothelial dysfunction assessed by flow-mediated dilation in the brachial artery demonstrated an attenuated response that was independent of obesity [43]. Children with HTN also demonstrate significantly narrower central retinal arteriolar diameters compared to normotensive peers [43]. Assessment of endothelial dysfunction using EndoPAT has yielded mixed results with limited utility in pediatric populations compared with other vascular assessment methods [44]. The ambulatory arterial stiffness index has been shown to correlate with pediatric HTN and its duration and etiology [45]. However, due to a lack of correlation with the gold-standard method for measurement of arterial stiffness (pulse wave velocity) the use of this methodology in pediatric practice remains limited [46]. The reported vascular changes in pediatric HTN are secondary to hemodynamic and biochemical stress on the endothelium which lead to reduced nitric oxide bioavailability and increased endothelin-1. These changes contribute to sustained endothelial dysfunction and vascular smooth muscle proliferation.

## 6. Blood Pressure Monitoring and Screening Guidelines

Current guidelines recommend annual BP screening in all children starting at 3 years of age [40]. However, the United States Preventive Services Task Force (USPSTF) reported that current evidence is insufficient to recommend routine BP screening [16]. The USPSTF concluded that evidence demonstrating the benefits of screening is insufficient and the primary factor driving this statement was related to the lack of adequate studies to demonstrate that screening will reduce risk of cardiovascular disease. This discrepancy largely reflects differences in methodology and evidentiary thresholds used to formulate recommendations. The level of evidence required by the USPSTF is difficult to obtain in pediatric HTN research. This is due to the long interval between exposure to elevated BP and the development of cardiovascular outcomes. However, the AAP extrapolates from biologically plausible mechanisms and recommends screening. Despite this controversy, most pediatricians continue to screen for elevated BP. However, it is important to note that the AAP guideline, endorsed by the American Heart Association (AHA), emphasizes the importance of standardized BP measurement and recommends ambulatory blood pressure monitoring (ABPM) to confirm the diagnosis [21]. In addition, this statement suggests 24 h ABPM to assess patients at higher risk of HTN such as those with chronic kidney disease, coarctation of the aorta, diabetes, OSA and genetic syndromes such as Williams syndrome, Turner syndrome, and neurofibromatosis type 1.

Building on these measurement strategies, the Study of High Blood Pressure in Pediatrics: Adult HTN Onset in Youth (SHIP AHOY) study demonstrated a risk-based definition of HTN in adolescents by examining the relationship between blood pressure and target organ damage [47]. This study demonstrated a dose–response relationship between BP and target organ damage with stronger associations observed for ABPM-derived measures [48].

## 7. Management and Interventions

Management of pediatric HTN starts with prevention. Multiple factors, as discussed above, are risk factors for pediatric HTN, and addressing them may delay or prevent its development. When HTN develops, the AAP recommends a stepwise approach to management that begins with lifestyle modifications, followed by pharmacologic therapy when indicated [40]. Lifestyle modification is an important part of management, and both dietary and behavioral factors influence BP. Management of obesity and sleep apnea is beneficial for controlling HTN. After a 6-month trial of non-pharmacologic therapy and management of comorbidities, pharmacologic treatment is typically initiated with a goal BP below the 90th percentile or 130/80 mmHg for children 13 years of age and older (whichever is lower). In children with symptomatic HTN, stage 2 HTN and comorbidities such as chronic kidney disease or diabetes, pharmacologic treatment is recommended in conjunction with lifestyle and dietary modification. More stringent BP targets are recommended for children with secondary HTN or high-risk conditions such as chronic kidney disease [49]. Pharmacologic management typically includes angiotensin-converting enzyme inhibitors, angiotensin receptor blockers, long-acting calcium channel blockers, or diuretics. Although some data on reversal of surrogate outcomes in pediatric HTN are available [35], evidence for long-term benefit is limited. Management recommendations in pediatric guidelines are predominantly extrapolated from adult data.

Studies assessing guideline adherence have demonstrated poor follow-up rates. These studies demonstrated that follow-up rates ranged from 10% to 17% among eligible patients. Centers with better resources had better follow-up rates [50,51]. These findings underscore the need for system-level interventions to improve adherence to follow-up protocols and optimize long-term outcomes. To that end, adolescents transitioning to adulthood should be enrolled in structured transition pathways to prevent loss of care continuity and therapeutic inertia during this high-risk period.

## 8. Future Directions

Despite significant advances in this field, studies standardizing treatment approaches and assessing the impact of treatment on long-term outcomes are needed. Prospective, longitudinal randomized studies incorporating frequent standardized BP assessments and treatment interventions are needed to help establish clinically meaningful BP thresholds for diverse pediatric populations and determine the effect of treatment on long-term cardiovascular outcomes. Furthermore, the contribution of potential confounders, including obesity and diabetes, to cardiovascular risk warrants careful evaluation. Cuffless devices for continuous BP monitoring are important innovations that may improve diagnosis and treatment. In addition, as discussed above, barriers to the implementation of guideline-directed therapy need to be further evaluated. This includes, but is not limited to, studies examining healthcare disparities, access to care, treatment adherence, and the transition from pediatric to adult healthcare services.

## 9. Conclusions

Pediatric HTN is prevalent and is associated with target organ damage. There is increasing evidence suggesting an association with cardiovascular outcomes. Timely diagnosis using ABPM, guided by refined screening guidelines, and comprehensive risk factor modification may potentially reverse early vascular injury and prevent adverse outcomes. Further studies can refine our approach and improve long-term cardiovascular outcomes.

## Data Availability

No new data were created or analyzed in this study.

## Abbreviations

The following abbreviations are used in this manuscript:

HTN	Hypertension
CVD	Cardiovascular Disease
BP	Blood Pressure
ABPM	Ambulatory Blood Pressure Monitoring
AAP	American Academy of Pediatrics
BMI	Body Mass Index
USPSTF	United States Preventive Services Task Force

## References

[1] Global Burden of Cardiovascular Diseases and Risks 2023 Collaborators (2025). Global, Regional, and National Burden of Cardiovascular Diseases and Risk Factors in 204 Countries and Territories, 1990–2023. J. Am. Coll. Cardiol..

[2] Blumenthal S., Epps R.P., Heavenrich R., Lauer R.M., Lieberman E., Mirkin B., Mitchell S.C., Boyar Naito V., O’Hare D., McFate Smith W. (1977). Report of the task force on blood pressure control in children. Pediatrics.

[3] Robinson C.H., Hussain J., Jeyakumar N., Smith G., Birken C.S., Dart A., Dionne J., Garg A., Kandasamy S., Karam S. (2024). Long-Term Cardiovascular Outcomes in Children and Adolescents with Hypertension. JAMA Pediatr..

[4] Zhou J., Shan S., Wu J., Song Y., Zhu L., Li Q., Zhang C., Zhu Y., Sheikh A., Rahimi K. (2026). Global prevalence of hypertension among children and adolescents aged 19 years or younger: An updated systematic review and meta-analysis. Lancet Child Adolesc. Health.

[5] Gartlehner G., Vander Schaaf E.B., Orr C., Kennedy S.M., Clark R., Viswanathan M. (2020). Screening for Hypertension in Children and Adolescents: Updated Evidence Report and Systematic Review for the US Preventive Services Task Force. JAMA.

[6] Faggiano A., Gherbesi E., Sala C., Carugo S., Grassi G., Cuspidi C., Tadic M. (2025). Elevated Blood Pressure and Cardiac Mechanics in Children and Adolescents: A Systematic Review and Meta-Analysis. Am. J. Hypertens..

[7] Rus R.R., Pac M., Obrycki Ł., Sağsak E., Azukaitis K., Sinha M.D., Jankauskiene A., Litwin M., HyperChildNet Working Group 3 (2023). Systolic and diastolic left ventricular function in children with primary hypertension: A systematic review and meta-analysis. J. Hypertens..

[8] Dogan K., Başar E.Z., Aytaç M.B., Şahin N., Bayrak Y.E., Bek K., Güngör H.S., Sönmez H.E., Babaoğlu K. (2024). Evaluation of endothelial dysfunction in hypertensive children and adolescents. Pediatr. Nephrol..

[9] Schweighofer N., Varda N.M., Caf P., Rupreht M., Kanic V., Brzan P.P. (2025). Assessment of epicardial adipose tissue volume and carotid intima-media thickness in children with primary arterial hypertension by magnetic resonance imaging. Radiol. Oncol..

[10] Schipper H.S., de Ferranti S. (2022). Atherosclerotic Cardiovascular Risk as an Emerging Priority in Pediatrics. Pediatrics.

[11] Ruan X., Zhu A., Wang T., Sun M., Chen K., Luo M., Li Z., Zou Q., Chen Y., Peng Y. (2025). Global Prevalence of Hypertension in Children and Adolescents Younger Than 19 Years: A Systematic Review and Meta-Analysis. JAMA Pediatr..

[12] Ouyang D., Cao W., Shen Y., Chen L., Hu G. (2025). Trends and risk factors of hypertension in US Children and Adolescents, 1999-2023. J. Hum. Hypertens..

[13] Rivier C.A., Renedo D.B., Sunmonu N.A., de Havenon A., Sheth K.N., Falcone G.J. (2024). Neighborhood Deprivation, Race, Ethnicity, and Undiagnosed Hypertension: Results from the All of Us Research Program. Hypertension.

[14] Suglia S.F., Koenen K.C., Boynton-Jarrett R., Chan P.S., Clark C.J., Danese A., Faith M.S., Goldstein B.I., Hayman L.L., Isasi C.R. (2018). Childhood and Adolescent Adversity and Cardiometabolic Outcomes: A Scientific Statement from the American Heart Association. Circulation.

[15] Meng Y., Sharman J.E., Iiskala F., Wu F., Juonala M., Pahkala K., Rovio S.P., Fraser B.J., Kelly R.K., Hutri N. (2025). Tracking and Transition Probability of Blood Pressure from Childhood to Midadulthood. JAMA Pediatr..

[16] Krist A.H., Davidson K.W., Mangione C.M., Barry M.J., Cabana M., Caughey A.B., Donahue K., Doubeni C.A., Epling J.W., US Preventive Services Task Force (2020). Screening for High Blood Pressure in Children and Adolescents: US Preventive Services Task Force Recommendation Statement. JAMA.

[17] Wen W., Psoter K.J., Solomon B.S., Urbina E.M., Brady T.M. (2024). Accuracy and Performance of Triage Blood Pressure Measurements in A Real-World Clinic Setting. J. Pediatr..

[18] Podoll A., Grenier M., Croix B., Feig D.I. (2007). Inaccuracy in pediatric outpatient blood pressure measurement. Pediatrics.

[19] Liu H., Zhao D., Sabit A., Pathiravasan C.H., Ishigami J., Charleston J., Miller E.R., Matsushita K., Appel L.J., Brady T.M. (2024). Arm Position and Blood Pressure Readings: The ARMS Crossover Randomized Clinical Trial. JAMA Intern. Med..

[20] Manzoli L., Simonetti V., D’Errico M.M., De Vito C., Flacco M.E., Forni C., La Torre G., Liguori G., Messina G., Mezzetti A. (2012). (In)accuracy of blood pressure measurement in 14 Italian hospitals. J. Hypertens..

[21] Flynn J.T., Urbina E.M., Brady T.M., Baker-Smith C., Daniels S.R., Hayman L.L., Mitsnefes M., Tran A., Zachariah J.P., Atherosclerosis, Hypertension, and Obesity in the Young Committee of the American Heart Association Council on Lifelong Congenital Heart Disease and Heart Health in the Young (2022). Ambulatory Blood Pressure Monitoring in Children and Adolescents: 2022 Update: A Scientific Statement from the American Heart Association. Hypertension.

[22] Flynn J.T., Khoury P.R., Ferguson M.A., Hanevold C.D., Lande M.B., Meyers K.E., Samuels J.A., Urbina E.M. (2025). Ambulatory Blood Pressure Phenotype and Cardiovascular Risk in Youth: The SHIP-AHOY Study. J. Pediatr..

[23] Swartz S.J., Srivaths P.R., Croix B., Feig D.I. (2008). Cost-effectiveness of ambulatory blood pressure monitoring in the initial evaluation of hypertension in children. Pediatrics.

[24] Chung J., Robinson C., Sheffield L., Paramanathan P., Yu A., Ewusie J., Sanger S., Mitsnefes M., Parekh R.S., Sinha M.D. (2023). Prevalence of Pediatric Masked Hypertension and Risk of Subclinical Cardiovascular Outcomes: A Systematic Review and Meta-Analysis. Hypertension.

[25] Jacobs D.R., Woo J.G., Sinaiko A.R., Daniels S.R., Ikonen J., Juonala M., Kartiosuo N., Lehtimäki T., Magnussen C.G., Viikari J.S.A. (2022). Childhood Cardiovascular Risk Factors and Adult Cardiovascular Events. N. Engl. J. Med..

[26] Freedman A.A., Perak A.M., Ernst L.M., Borders A., Miller G.E., Allen N.B., Gilman S.E., Khan S.S. (2025). High Blood Pressure in Childhood and Premature Cardiovascular Disease Mortality. JAMA.

[27] Yang L., Sun J., Zhao M., Liang Y., Bovet P., Xi B. (2020). Elevated blood pressure in childhood and hypertension risk in adulthood: A systematic review and meta-analysis. J. Hypertens..

[28] Iiskala F., Meng Y., Juonala M., Magnussen C.G., Pahkala K., Hutri N., Kähönen M., Lehtimäki T., Fogelholm M., Jokinen E. (2026). Life-Course Blood Pressure Levels, 38-Year Tracking, and Prediction of Hypertension. J. Am. Heart Assoc..

[29] Franco O.H., Peeters A., Bonneux L., de Laet C. (2005). Blood pressure in adulthood and life expectancy with cardiovascular disease in men and women: Life course analysis. Hypertension.

[30] Du T., Fernandez C., Barshop R., Chen W., Urbina E.M., Bazzano L.A. (2019). 2017 Pediatric Hypertension Guidelines Improve Prediction of Adult Cardiovascular Outcomes. Hypertension.

[31] Chung J., Robinson C.H., Yu A., Bamhraz A.A., Ewusie J.E., Sanger S., Mitsnefes M., Parekh R.S., Raina R., Thabane L. (2023). Risk of Target Organ Damage in Children With Primary Ambulatory Hypertension: A Systematic Review and Meta-Analysis. Hypertension.

[32] Sinha M.D., Azukaitis K., Sladowska-Kozłowska J., Bårdsen T., Merkevicius K., Sletten I.S.K., Obrycki Ł., Pac M., Fernández-Aranda F., Bjelakovic B. (2022). Prevalence of left ventricular hypertrophy in children and young people with primary hypertension: Meta-analysis and meta-regression. Front. Cardiovasc. Med..

[33] Zhang T., Li S., Bazzano L., He J., Whelton P., Chen W. (2018). Trajectories of Childhood Blood Pressure and Adult Left Ventricular Hypertrophy: The Bogalusa Heart Study. Hypertension.

[34] Devereux R.B., Wachtell K., Gerdts E., Boman K., Nieminen M.S., Papademetriou V., Rokkedal J., Harris K., Aurup P., Dahlöf B. (2004). Prognostic significance of left ventricular mass change during treatment of hypertension. JAMA.

[35] Kupferman J.C., Paterno K., Mahgerefteh J., Pagala M., Golden M., Lytrivi I.D., Ramaswamy P. (2010). Improvement of left ventricular mass with antihypertensive therapy in children with hypertension. Pediatr. Nephrol..

[36] Genovesi S., Tassistro E., Giussani M., Antolini L., Lieti G., Orlando A., Montemerlo M., Patti I., Parati G. (2023). Association between lifestyle modifications and improvement of early cardiac damage in children and adolescents with excess weight and/or high blood pressure. Pediatr. Nephrol..

[37] Shilly S., Merchant K., Singer P., Frank R., Gurusinghe S., Infante L., Sethna C.B. (2019). Left ventricular cardiac geometry and ambulatory blood pressure in children. J. Clin. Hypertens..

[38] Richey P.A., Disessa T.G., Somes G.W., Alpert B.S., Jones D.P. (2010). Left ventricular geometry in children and adolescents with primary hypertension. Am. J. Hypertens..

[39] Ezon D., Duong S.Q., Stoffels G., Lopez L., Mahgerefteh J. (2025). Height-Based Pediatric Echocardiographic Z Scores Are Valid in Patients With Normal Body Mass Index and May Be Advantageous in Obese Patients. J. Am. Soc. Echocardiogr..

[40] Flynn J.T., Kaelber D.C., Baker-Smith C.M., Blowey D., Carroll A.E., Daniels S.R., de Ferranti S.D., Dionne J.M., Falkner B., Flinn S.K. (2017). Clinical Practice Guideline for Screening and Management of High Blood Pressure in Children and Adolescents. Pediatrics.

[41] Kamsheh A.M., Meyers K.E., Palermo R.A., Wu L., Burstein D.S., Edelson J.B., Lin K.Y., Maeda K., Rossano J.W., Wittlieb-Weber C.A. (2024). Hypertension: An Important But Reversible Cause of Systolic Dysfunction in a Cohort of Pediatric Patients. Pediatr. Cardiol..

[42] Litwin M., Niemirska A., Sladowska-Kozlowska J., Wierzbicka A., Janas R., Wawer Z.T., Wisniewski A., Feber J. (2010). Regression of target organ damage in children and adolescents with primary hypertension. Pediatr. Nephrol..

[43] Kos M., Nađ T., Stupin A., Drenjančević I., Kolobarić N., Šušnjara P., Mihaljević Z., Damašek M., Pušeljić S., Jukić I. (2024). Juvenile primary hypertension is associated with attenuated macro- and microvascular dilator function independently of body weight. J. Hypertens..

[44] Hayden J., O’Donnell G., deLaunois I., O’Gorman C. (2023). Endothelial Peripheral Arterial Tonometry (Endo-PAT 2000) use in paediatric patients: A systematic review. BMJ Open.

[45] Simonetti G.D., VON Vigier R.O., Wühl E., Mohaupt M.G. (2008). Ambulatory arterial stiffness index is increased in hypertensive childhood disease. Pediatr. Res..

[46] Stergiou G.S., Kollias A., Giovas P.P., Papagiannis J., Roussias L.G. (2010). Ambulatory arterial stiffness index, pulse pressure and pulse wave velocity in children and adolescents. Hypertens. Res..

[47] Mendizabal B., Urbina E.M., Becker R., Daniels S.R., Falkner B.E., Hamdani G., Hanevold C.D., Hooper S.R., Ingelfinger J.R., Lande M. (2018). SHIP-AHOY (Study of High Blood Pressure in Pediatrics: Adult Hypertension Onset in Youth). Hypertension.

[48] Hamdani G., Urbina E.M., Daniels S.R., Falkner B.E., Ferguson M.A., Flynn J.T., Hanevold C.D., Ingelfinger J.R., Khoury P.R., Lande M.B. (2025). Youth Blood Pressure and Target Organ Injury Markers: The SHIP AHOY Study. Hypertension.

[49] Chanchlani R., Brady T., Kruger R., Sinha M.D. (2026). Under pressure: The lifelong cardiovascular health of children and youth with primary hypertension. Lancet Child Adolesc. Health.

[50] Carroll A.J., Tedla Y.G., Padilla R., Jain A., Segovia E., Moin A., Wallace A.S., Sanuade O.A., Langman C.B., Mohanty N. (2023). Adherence to the 2017 Clinical Practice Guidelines for Pediatric Hypertension in Safety-Net Clinics. JAMA Netw. Open.

[51] Rea C.J., Brady T.M., Bundy D.G., Heo M., Faro E., Giuliano K., Goilav B., Kelly P., Orringer K., Tarini B.A. (2022). Pediatrician Adherence to Guidelines for Diagnosis and Management of High Blood Pressure. J. Pediatr..

